# Identification of the ESKAPE pathogens by mass spectrometric analysis of microbial membrane glycolipids

**DOI:** 10.1038/s41598-017-04793-4

**Published:** 2017-07-25

**Authors:** Lisa M. Leung, William E. Fondrie, Yohei Doi, J. Kristie Johnson, Dudley K. Strickland, Robert K. Ernst, David R. Goodlett

**Affiliations:** 1 Department of Microbial Pathogenesis, University of Maryland, Baltimore, MD 21201 USA; 2 Center for Vascular and Inflammatory Diseases, University of Maryland, Baltimore, MD 21201 USA; 3 Division of Infectious Diseases, University of Pittsburgh, Pittsburgh, PA 15261 USA; 4 Department of Pathology, University of Maryland, Baltimore, MD 21201 USA; 5 Department of Pharmaceutical Sciences, University of Maryland, Baltimore, MD 21201 USA

## Abstract

Rapid diagnostics that enable identification of infectious agents improve patient outcomes, antimicrobial stewardship, and length of hospital stay. Current methods for pathogen detection in the clinical laboratory include biological culture, nucleic acid amplification, ribosomal protein characterization, and genome sequencing. Pathogen identification from single colonies by matrix-assisted laser desorption/ionization time-of-flight mass spectrometry (MALDI-TOF-MS) analysis of high abundance proteins is gaining popularity in clinical laboratories. Here, we present a novel and complementary approach that utilizes essential microbial glycolipids as chemical fingerprints for identification of individual bacterial species. Gram-positive and negative bacterial glycolipids were extracted using a single optimized protocol. Extracts of the clinically significant ESKAPE pathogens: *E* *nterococcus faecium*, *S* *taphylococcus aureus*, *K* *lebsiella pneumoniae*, *A* *cinetobacter baumannii*, *P* *seudomonas aeruginosa*, and *E* *nterobacter* spp. were analyzed by MALDI-TOF-MS in negative ion mode to obtain glycolipid mass spectra. A library of glycolipid mass spectra from 50 microbial entries was developed that allowed bacterial speciation of the ESKAPE pathogens, as well as identification of pathogens directly from blood bottles without culture on solid medium and determination of antimicrobial peptide resistance. These results demonstrate that bacterial glycolipid mass spectra represent chemical barcodes that identify pathogens, potentially providing a useful alternative to existing diagnostics.

## Introduction

Infectious diseases pose an ongoing threat to public health. Rapid and accurate pathogen detection is needed to guide physicians in the treatment of infection to improve patient and economic outcomes^1^. In the clinical laboratory, bacterial isolates are routinely identified by morphological and biochemical methods. Once the bacteria are identified, additional testing such as antibiotic susceptibility can be performed to guide definitive antibiotic treatment. Microbiological culture followed by biochemical identification of bacteria is the current gold standard for clinical diagnostics, but this strategy requires additional testing to detect closely related organisms, as with the Enterobacteriaceae^2^. Nucleic acid amplification and sequencing of essential bacterial genes, such as the 16S rDNA, for bacterial identification offers increased accuracy; however, high sensitivity can result in false positives and it may not provide valuable sub-species information^3^. Collectively, these methods are time intensive, require at least 24 hours of incubation of clinically obtained material, and often significantly increase the cost and burden of diagnostic laboratory support. Next generation sequencing of whole bacterial genomes is proposed as a culture-free alternative, but it is highly technical with regard to bioinformatic interpretation and is more costly compared to traditional methods^4^.

To address some of the current challenges in bacterial identification, mass spectrometric analysis of bacterial proteins is emerging as the dominant technology in many clinical laboratories. Currently, there are two commercially available platforms, the Bruker MALDI Biotyper^5^ and bioMérieux VITEK MS^6, 7^. These platforms identify bacteria by comparison of a mass spectrum of bacterial proteins from an unknown species to a reference library of previously recorded mass spectra^5–8^. These protein-based platforms have significant limitations, including: requirement for cell culture to obtain pure colonies, poor identification at the species level for closely related species (e.g. *Escherichia coli v*. *Shigella flexneri*), inability to identify pathogens directly from complex biological samples, and inability to identify antimicrobial resistance on the FDA-approved platform although Bruker has introduced a workflow for the identification of β-lactamases on the research use only (RUO) platform^9^. To address the urgent need for novel technologies that can overcome these challenges and expand the ability to rapidly diagnose bacterial infections, we propose the use of highly abundant membrane glycolipids as another class of molecules to exploit for bacterial identification.

Bacterial membranes are composed of lipids of diverse structure and composition. Similar to eukaryotic cell membranes, microbial membranes are composed of a bilayer of amphiphilic glycerophospholipids. In Gram-negative bacteria, there are two distinct membranes separated by a periplasm, whereas in Gram-positive bacteria, the membrane is enclosed by a cell wall^10^. Previously, use of bacterial membrane phospholipids had been proposed to phenotype bacteria^2^. Specifically, analysis of fatty acids and membrane phospholipids by gas chromatography with flame ionization detection or mass spectrometry (MS) was explored with limited success. Because fatty acid profiles are not unique for each microbial species, species are differentiated via differences in ion intensities, which vary as a function of growth^11–13^. This is further complicated by the fact that bacteria and mammalian hosts share some of the same phospholipids; consequently, direct analysis of patient specimens is not possible due to the inability to distinguish bacterial phospholipids from those of the host.

Alternatively, microbial membranes possess more complex, glycosylated lipids that are exclusive to bacterial cell membranes and thus are not produced by mammals. They are present in high abundance, approximately 10^6^ molecules per bacterium, and are readily extracted from bacteria grown under laboratory conditions or directly from biological fluids. In Gram-negative bacteria, lipopolysaccharide (LPS) comprises the outer leaflet of the outer membrane. The general architecture consists of a lipophilic anchor moiety (lipid A), a core oligosaccharide of arranged hexoses, and an O-polysaccharide chain of repeating subunits. The endotoxin component, lipid A (LA), consists of a glucosamine disaccharide backbone flanked by terminal phosphate residues and fatty acyl chains that extend from the backbone^14^. In contrast, Gram-positive bacteria have numerous cell wall glycans including cardiolipin and lipoteichoic acid (LTA), which is composed of a diacylglycerol (DAG) lipid that anchors in the membrane and a complex oligosaccharide that penetrates the cell wall^15^. Examination of the literature suggests these bacterial glycolipids could provide species-specific mass spectral profiles due to their immense diversity in the arrangement of fatty acyl side chains and sugar-associated functional groups^16, 17^. We thus hypothesized that these glycolipids represent novel chemical fingerprints that would enable identification of bacteria by MS in a manner similar to bacterial proteins.

To evaluate the diagnostic potential of these membrane glycolipids, we examined the ESKAPE pathogens: Gram-positive *E* *nterococcus faecium* and *S* *taphylococcus aureus* and Gram-negative *K* *lebsiella pneumoniae*, *A* *cinetobacter baumannii*, *P* *seudomonas aeruginosa*, and *E* *nterobacter* spp., so named for their ability to escape antibiotic treatment. They are of considerable concern due to their prevalence in hospital-acquired infections and acquisition of resistance to antibiotics such as polymyxins^18^, a family of cationic antimicrobial peptides (CAMPs) available as polymyxin B sulfate and colistin (polymyxin E). CAMPs bind negatively-charged LPS through electrostatic interactions with the terminal phosphate groups and insert in the bacterial membrane, disrupting permeabilization leading to cell death^19^. Emergence of polymyxin resistance has been observed in the ESKAPE pathogens *K*. *pneumoniae* ^20^, *A*. *baumannii* ^21^, and *P*. *aeruginosa* ^22^. When analyzed by MS, we and others have shown that antimicrobial-resistant ESKAPE isolates are chemically distinct from their susceptible counterparts illustrating the potential of our approach to not only identify bacteria but also to improve antibiotic stewardship by reducing the use of broad spectrum antibiotics.

In this study, we utilize bacterial glycolipid extracts in a manner analogous to the protein-based platform to identify the ESKAPE pathogens by MALDI-TOF-MS. Using a single extraction protocol originally designed for LPS extraction, we were able to differentiate all ESKAPE pathogens by dot product analyses of their mass spectra. Furthermore, a glycolipid mass spectral library containing 50 unique microbial entries including the ESKAPE pathogens was built using the database and software package of the MALDI Biotyper, which is a platform currently in use in the clinical laboratory. Importantly for clinical use, the glycolipid-based method has an advantage over the protein-based approach: antimicrobial resistant strains could be distinguished from the related susceptible strains in tested cases. These results suggest developing MS glycolipid profiling as a diagnostic platform that could impact clinical practice.

## Results

### Glycolipid mass spectral library construction

**Figure 1:**
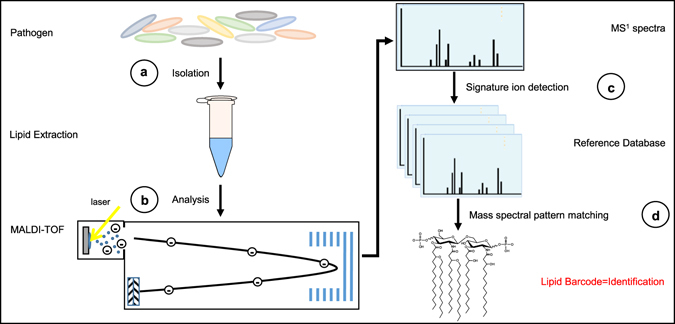
Strategy for glycolipid-based mass spectrometry platform for pathogen identification. (**a**) Microbes are isolated from pure culture or biological specimen and whole cell lipids are extracted by hot ammonium-isobutyrate (**b**) Lipid extracts are purified and analyzed by MALDI-TOF-MS (**c**) A mass spectrum of membrane glycolipids is acquired and compared against an extensive reference database of mass spectral profiles from known organisms via pattern-matching to (**d**) Generate a digital identification output and an assigned confidence score.

For generation of high quality mass spectra for inclusion in the library, samples were grown in liquid culture and lipids extracted as described by El Hamidi *et al*.^23^. This small-scale ammonium isobutyrate extraction disrupts the membrane and liberates the glycolipids (LA, LTA, and DAG) from their polysaccharide constituents. To account for previously observed differences in glycolipid structure when growth temperature is altered, bacteria were grown at both 25 °C and 37 °C and their extracts were analyzed by MALDI-TOF-MS in negative ion mode over a limited mass range *m/z* 1,000–2,400 using the matrix norharmane^24^. The strategy that allowed for the generation of an optimized mass spectral library consisting of a minimum of twelve unique strains per ESKAPE pathogen is outlined in Fig. 1. Supplementary Table S1 lists the individual strains used in this study. This process resulted in a mass spectral ESKAPE pathogen library of six unique species and an additional 44 non-ESKAPE pathogens used as decoys for further analyses (Supplementary Table S2).

### Lipid extraction and MALDI-TOF-MS reproducibility

**Figure 2:**
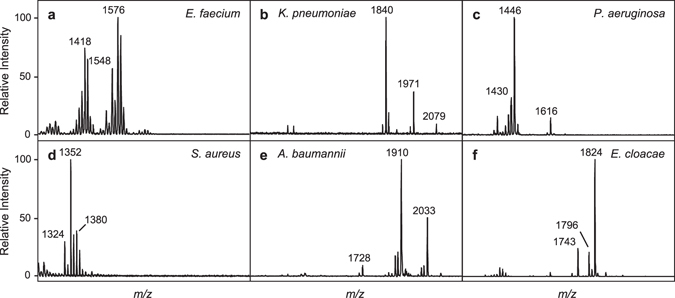
Representative mass spectra from ESKAPE pathogens: (**a**) *Enterococcus faecium*; (**b**) *Klebsiella pneumoniae*; (**c**) *Pseudomonas aeruginosa*; (**d**) *Staphylococcus aureus*; (**e**) *Acinetobacter baumannii*; and, (**f**) *Enterobacter cloacae. m/z* values of select ions are given.

To evaluate the reproducibility of mass spectra produced by analysis of extracts between different glycolipid extractions and cultures, five independent biological cultures of *P*. *aeruginosa* were grown daily (intra-day) over five consecutive days (inter-day) producing a total of 25 mass spectra. In addition, 30 technical extraction replicates were generated from a single culture and processed on the same day. A single mass spectrum from each sample was recorded per day. Under consistent culture, extraction, and analysis conditions, similar profiles were produced with the expected *P*. *aeruginosa* signature ions at *m/z* 1430, 1446, and 1616 (Supplementary Fig. S1). As shown in Fig. 2, signature ions are defined as those ions that are unique to a given species. The mass spectra were combined to generate an MSP (Main SPectra), which is a projection of multiple mass spectra used by the MALDI Biotyper, and added to the library containing MSPs from 50 unique microorganisms including the ESKAPE pathogens within the Biotyper software package. Using the Biotyper’s software platform, each individual mass spectrum was then compared against the library of summed mass spectra. Confidence scores, which reflect the strength of the identification, were subsequently determined with a mean log confidence score of 2.87 ± 0.014 (mean ± variance) for intra-/inter-day variability and 2.86 ± 0.004 (mean ± variance) for 30-replicate variability (Supplementary Fig. S1). These values are comparable to Bruker’s protein-based scores that advise any score between 2.0 and 3.0 as a high positive species identification probability.

### Species-specific signature ions differentiate all ESKAPE pathogens

**Figure 3:**
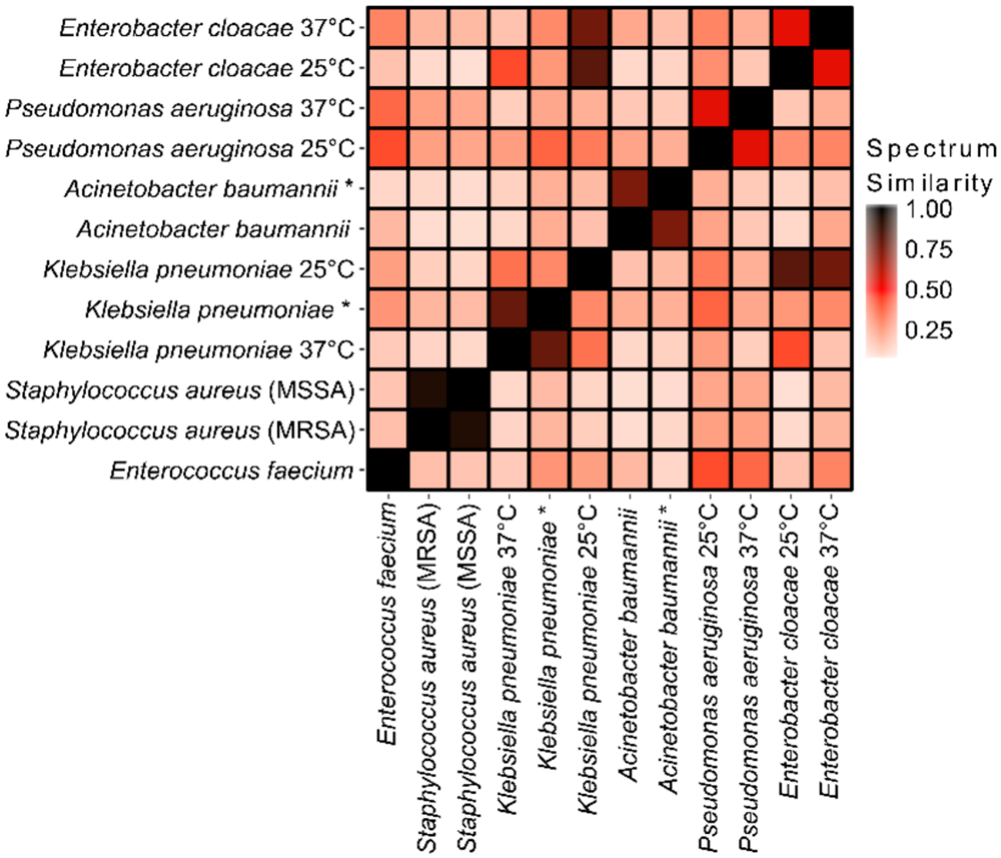
Dot product analysis of mass spectra for differentiation of ESKAPE pathogens. Mass spectra were acquired from lipid extracts of each ESKAPE pathogen. Species were compared by calculating a pairwise dot product between mass lists of ions from each mass spectrum, a measure of spectrum similarity. A similarity score of 1.0 is an identical match (black squares). White squares represent a score of 0.0 where there is no match. (*) indicate colistin-resistant strains.

To objectively determine whether the observed differences between mass spectra for all ESKAPE pathogens (Fig. 2) would permit differentiation, we carried out pair-wise dot product analyses of all ESKAPE mass spectra^25^. A minimum signal-to-noise of 8.0 (optimized to ensure only *bona fide m/z* values for glycolipid ions were considered) was used to cull low quality mass spectra with the remainder being assembled into lists of ions containing *m/z* and normalized ion intensity for each mass spectrum. The dot product between pairs of ion lists was calculated as a measure of similarity between mass spectra. This resulted in a mass spectral similarity score between 0 (least similar) and 1 (identical), shown as a heat map of representative strains in Fig. 3 with similarity scores listed in Supplementary Table S3. In addition to the ability to distinguish all ESKAPE species from one another, glycolipid mass spectra were able to distinguish *K*. *pneumoniae*, *P*. *aeruginosa*, and *E*. *cloacae* grown at 25 and 37 °C, temperatures that mimic insect and mammalian growth conditions, respectively. As an example, we observed a shift in the base peak from *m/z* 1796 to 1840 for *K*. *pneumoniae* strains grown at 37 °C versus those grown at 25 °C (Supplementary Fig. S2), which is known to result from an exchange of a laurate (C12:0) for a myristate (C14:0) at the C-2′ position of the glucosamine backbone and a hydroxylation of the 2′ acyl-oxo-acyl group^26^. Supplementary Fig. S3 provides a more comprehensive dot product comparison with ten strains per ESKAPE pathogen. Hierarchical clustering further demonstrates the ability to differentiate between the individual ESKAPE pathogens by their glycolipid mass spectra. In addition, *K*. *pneumoniae* and *A*. *baumannii* strains with resistance to colistin clustered together, illustrating the potential to distinguish resistant isolates.

### Detection of antimicrobial resistance

**Figure 4:**
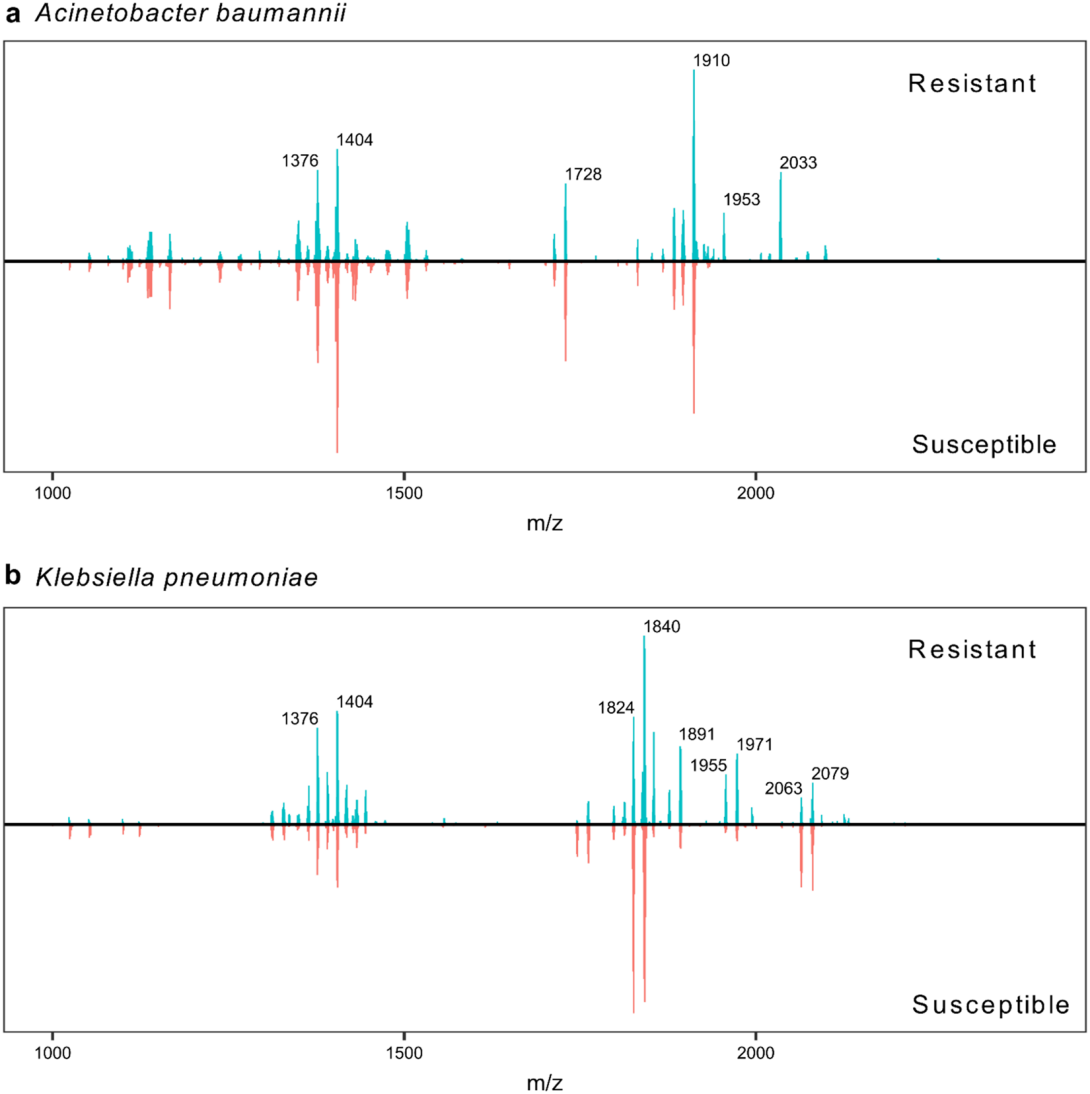
Consensus mass spectra were created by summation of detected ions from all replicates of colistin-resistant (top panels) and colistin-susceptible (bottom panels) *A*. *baumannii* (**a**) and *K*. *pneumoniae* (**b**). The mirrored consensus mass spectra highlight ions of core lipid A structures for *A*. *baumannii* (*m/z* 1376, 1404, 1728, 1910) and *K*. *pneumoniae* (*m/z* 1376, 1404, 1824, 1840, 2063, 2079). Importantly, these consensus spectra reveal consistent diagnostic ions for detecting colistin resistance in *A*. *baumannii* (*m/z* 1953, 2033) and *K*. *pneumoniae* (*m/z* 1891, 1955, 1971).

To further assess the ability of our approach to identify resistance patterns to CAMPs, we examined clinical isolates of *K*. *pneumoniae* (60 clinical isolates) and *A*. *baumannii* (213 clinical isolates) obtained from patients admitted to the University of Pittsburgh Medical Center. For this analysis, colistin-susceptible isolates were determined to have a minimum inhibitory concentration (MIC) ≤ 2 µg/mL colistin; resistant strains were determined by an MIC ≥ 4 µg/mL according to recommendations of the Clinical and Laboratory Standards Institute^27^. The colistin-resistant *A*. *baumannii* strain shows an additional ion at *m/z* 2033 (summation of detected mass peaks in Fig. 4a and representative mass spectra in Supplementary Fig. S4), reflecting an ethanolamine addition onto the terminal phosphate of *m/z* 1910 (Supplementary Fig. S4). In Fig. 4b and Supplementary Fig. S4, colistin-resistant *K*. *pneumoniae* strains clearly show unique ions at *m/z* 1955 and 1971 that are not present in susceptible strains, a mass shift caused by an aminoarabinose addition to the hexa-acylated LA structures at *m/z* 1824 and 1840. Ions at *m/z* 1953 and 1891 result from a difference of one phosphate moiety (∆ 80 Da) from the modified lipid A structures at *m/z* 2033 and 1971 respectively, and occur either from monophosphorylated lipid A residing in the membrane or degradation during extraction; these ions are also considered markers of resistance^21, 28^. Consequently, there is a high positive correlation between these ions and resistance: when all library mass spectra were examined, we found that the presence of resistance ions (above a signal-to-noise ratio of 8) was detected by MS in 97.3% of *K*. *pneumoniae* and 88.9% of *A*. *baumannii* resistant isolates (as determined by MIC). Furthermore, we used dot product as a means to distinguish colistin susceptibility. Detected mass peaks from 50% of all mass spectral replicates were summed to generate a consensus spectrum for each phenotype (*A*. *baumannii v*. *K*. *pneumoniae*, susceptible *v*. resistant), and dot products were calculated between the remaining test set of mass spectra and the consensus spectrum in that phenotype (Supplementary Fig. S5). In general, mass spectra tended to achieve a higher similarity score when compared to the consensus spectrum with concurrent susceptibility; this is particularly evident for the *K*. *pneumoniae* strains. Finally, this platform was adapted to an existing clinical diagnostic, the MALDI Biotyper informatics package. All mass spectra from similar groups (susceptible and resistant) for these two species were assembled into MSPs. A randomized 30% of mass spectra were designated, and identifications were scored against the remaining 70% in addition to 50 organisms in the library (Supplementary Table S4). Colistin-resistant strains of *K*. *pneumoniae* and *A*. *baumannii* were identified with accuracy rates of 69% and 92, respectively, for correctly predicting colistin susceptibility in tested strains and 88% and 92% positive species identification overall.

### Identification from polymicrobial mixtures

In order to highlight the potential of our method to analyze polymicrobial mixtures, mass spectra from samples containing three ESKAPE pathogens, *S*. *aureus*, *K*. *pneumoniae*, and *P*. *aeruginosa*, often found together in polymicrobial infections^29^, were generated. Mass spectra, including those from either co-cultured organisms or those produced by mixing extracts post-culture of individual organisms, allowed each of the three species to be visually identified based on detection of each species’ signature ions (Supplementary Fig. S6).

### Direct identification from blood culture

**Figure 5:**
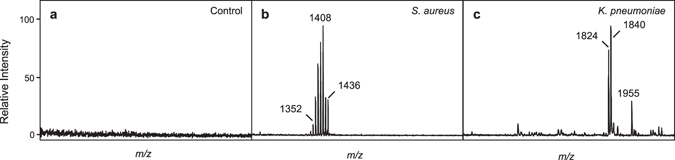
Detection of *S*. *aureus* and *K*. *pneumoniae* from blood culture. (**a**) Blood culture control containing sterile blood (**b**) Blood culture containing MRSA M2 after overnight growth (24 hours), and (**c**) Blood culture containing *K*. *pneumoniae* B6 after six hours growth. A 10^4^ CFU inoculate was seeded into 10 mL blood, transferred to standard aerobic culture bottles and sampled at 1, 2, 4, 6, and 24 hours. Differential centrifugation allowed separation of human cells. Extraction and mass analysis was performed.

In order to demonstrate applicability of our platform to relevant clinical samples, *K*. *pneumoniae* and *S*. *aureus* were cultivated in blood culture (BC) bottles (to which sterile blood had been added) at an initial inoculum of 10^3^ CFU/mL. We achieved detection from this initial inoculum after six hours for *K*. *pneumoniae* and after 24 hours for *S*. *aureus* (Fig. 5). Figure 5a shows a mass spectrum of a sterile BC control demonstrating that there are minimal background contaminants within the *m/z* range of these glycolipids of interest. Samples were compared against the Biotyper glycolipid library, and positive identifications were determined to be the top-scoring organism with a confidence log score >1.7 as throughout the study. The mass spectrum from *K*. *pneumoniae* in blood culture was correctly identified as *K*. *pneumoniae* with a score of 2.155. The *S*. *aureus* mass spectrum was positively identified at the genus level, but at the species level as *Staphylococcus haemolyticus*. This was attributable to a change in the signature ions after growth in blood bottles (Supplementary Fig. S7) that was consistent when multiple *S*. *aureus* strains were tested, thereby representing a unique phenotype for this bacterium in the context of a blood infection that will be included in future libraries. When a novel MSP was generated for *S*. *aureus* in blood culture, test mass spectra were positively identified as *S*. *aureus* with a score of 2.467. Currently, the limit of detection (LOD) of these bacteria in blood bottles is 10^8^ CFU/mL whereas the LOD from cells grown in pure laboratory culture is 10^5^ CFU/mL.

## Discussion

Infectious diseases have a significant global health impact. Conventional diagnostics involving microbiological, biochemical, and molecular assays typically require days or even weeks for fastidious organisms to undertake, often yielding inaccurate or incomplete diagnoses^2, 30–32^. This can have devastating consequences during an infection; therefore, innovative technologies are urgently needed to improve patient outcomes. In this study, we investigated a novel approach to bacterial identification, namely, analysis of microbial membrane glycolipids by MALDI-TOF-MS. Prior research has shown that complex glycolipids found in high abundance in bacterial membranes exhibit species-specific structural characteristics that present “signature ions” unique to a given species. We hypothesized that mass spectrometric analysis of these glycolipid fingerprints could differentiate and identify bacterial species as well as provide practical sub-species information. To demonstrate the validity of this approach, a library of glycolipid mass spectra was built representing 35 Gram-negative bacteria, 11 Gram-positive bacteria, and 4 fungal species. Glycolipid mass spectra were shown to be highly reproducible between mass spectral replicates. Furthermore, all ESKAPE species produced unique profiles visually but also objectively by dot product analysis.

Mass spectrometric phenotyping of bacterial proteins for identification exists on two commercial systems, the Bruker MALDI Biotyper (Bruker Daltonics Inc., Billerica MA) and bioMérieux VITEK MS (bioMérieux S.A., France). Both identify organisms via pattern-matching of a sample mass spectrum to a reference database of known organisms, and both achieve accuracy of ~90% for organisms in their clinical platforms. In the most recent study comparing them^8^, study authors determined accuracy rates of 86.4% for the Biotyper and 92.3% for the VITEK MS for correctly identifying a diverse range of microorganisms. This is comparable to our results using Biotyper (Supplementary Table S4) although accuracy rates of these platforms improve for routinely isolated strains^30^ or strains for which specialized databases exist on these platforms^6, 7^. Limitations to the capabilities of these platforms include identification of antibiotic resistance, identification from complex samples like blood, and identification from polymicrobial samples^9^. We successfully used our approach to solve these three unmet needs from the protein-based phenotyping approach. Importantly, we were able to adapt the existing Biotyper software to recognize glycolipid mass spectra and make species identifications as well as distinguish colistin-susceptible from resistant *K*. *pneumoniae* and *A*. *baumannii*, suggesting an immediate use for our approach that could augment protein-based identifications that fail to distinguish antibiotic resistance strains.

Most Gram-negative bacteria are susceptible to polymyxins (including colistin), but emergence of resistant isolates is increasing. Transfer of neutral or positively-charged functional groups onto LA are believed to decrease its electronegativity and protect it from attack by polymyxins, as well as host antimicrobial peptides^19^. As observed with *K*. *pneumoniae* and *A*. *baumannii*, this well-characterized adaptation produces large shifts in *m/z* values that are readily identified by MALDI-TOF-MS, and we have shown a high positive correlation between these mass spectral profiles and incidence of resistance. While we achieved compelling accuracy rates on the Biotyper platform and demonstrated adaptability of our platform to a commercially available diagnostic, they do not fully reflect how different the glycolipid mass spectra are due to the manner in which the Biotyper makes an identification, necessitated by the complexity of a bacterial protein spectrum. Continuing development of algorithms specific to our glycolipid approach promise to improve the performance of the platform both for identification and susceptibility prediction, as demonstrated by manual selection of colistin resistance associated peaks and calculation dot products against consensus mass spectra.

Our glycolipid-based approach to bacterial identification was also able to identify bacteria after culture in blood culture bottles, a clinically relevant source where identification of organisms is crucial in guiding appropriate therapy. During a suspected bloodstream infection, patient blood samples are introduced directly into a blood culture identification system. A sensor in the bottle indicates when the culture is positive and can be processed by downstream diagnostics, a process that can take 72 hours or longer^33^. Our study shows that *S*. *aureus* and *K*. *pneumoniae* cultured in blood bottles produced mass spectra that uniquely identifies them with the Biotyper software package, albeit with a shift in the signature ions observed for *S*. *aureus*. Importantly, the clean mass spectrum baseline from the control blood culture bottle illustrates an important point for analysis of human clinical samples, namely that the lipid extraction process produces mass spectra devoid of host lipids in the *m/z* range examined. This fact allows identification directly from blood culture, hours earlier than other methods. The estimated LOD for detecting bacterial glycolipids directly from blood bottle culture was 10^8^ cells, which is presently higher than the bacterial concentration of 10^1^ to 10^3^ CFU/mL that typically occurs in septic patients. This means our current method requires some degree of amplification (6 to 24 hours in the present study) for clinical use. However, we believe that optimization through refinement of extraction and sample preparation will further reduce amplification and processing time with the ultimate goal of detection of infectious agents directly from biological fluids with no amplification by our method.

Mass spectrometry of microbial lipids has been previously explored as a diagnostic through hydrolysis and analysis of total membrane fatty acids. Recent renewed interest has focused on analysis of the complex glycolipids used here, which are exclusive to microbial membranes and found in a higher *m/z* range than most host phospholipids and fatty acids produced by hydrolysis of LA and LTA. For example, others have reported detection of LA via MALDI-TOF by direct analysis of bacterial colonies^34^ and by use of rapid evaporative ionization mass spectrometry (REIMS) of select colonies^35^, the latter being an ambient ionization method involving electrical current applied directly to the surface of an analyte (in this case a colony) at the inlet of the mass analyzer. While these two reports offer direct analysis from pure colonies, our strategy uses an extraction step to isolate glycolipids of interest that can be done directly from biological fluids such as blood, urine, and wound effluent. While our less direct method could be seen as a disadvantage, our results produce higher quality mass spectra devoid of host lipids, which in turn produces higher confidence scores. Furthermore, we are working to develop an extraction protocol that will appreciably reduce the time for sample preparation to less than one hour from start to finish, improve LOD, and ultimately bypass the need for culture.

In conclusion, our work presents a novel diagnostic approach for identifying harmful pathogens as well as common organisms that can offer a complementary approach with added value to existing clinical protein-based platforms to strengthen the overall diagnostic power of a clinical lab.

## Materials and Methods

### Selection of strains

Strains were chosen in consultation with and individual clinical isolates were obtained through established collaborations with our infectious disease collaborators, Drs. Yohei Doi (University of Pittsburgh) and J. Kristie Johnson (University of Maryland). *S*. *aureus* strains were obtained from Dr. Mark Shirtliff (University of Maryland). All protocols were approved by the institutional review boards of the University of Pittsburgh (IRB #: PRO12060302) and the University of Maryland – Baltimore (IRB #: HP-00041044). Informed consent was not obtained since study involves de-identified, leftover biological specimens from standard patient care. Strains were identified by standard clinical laboratory methods including microbiological culture and confirmed by MS protein typing. A full list of strains can be found in Supplementary Table S1 to include 12 strains of *E*. *faecium*, 19 strains of *S*. *aureus*, 60 strains of *K*. *pneumoniae*, 213 strains of *A*. *baumannii*, 15 strains of *P*. *aeruginosa*, and 13 strains of *E*. *cloacae*. Within species, strains with different antimicrobial susceptibility were evaluated where there is a high prevalence of resistance. MICs for colistin were determined by broth dilution, agar dilution, and/or ETEST (bioMérieux S.A., France) using recommendations stipulated by the Clinical and Laboratory Standards Institute breakpoints for *E*. *coli* ATCC 25922 and *P*. *aeruginosa* ATCC 27853^27^.

### Analysis from blood culture bottles

Sterile blood was obtained from the University of Maryland Medical Center (UMMC) blood bank and stored at 4 °C upon receipt. Blood (5–10 mL) was inoculated into BACTEC™ blood culture bottles (Becton Dickinson, Sparks MD) according to manufacturer’s recommendations. Strains were seeded into blood bottles at 10^1^ to 10^8^ CFU. These were sampled immediately and incubated at 37 °C with shaking then sampled every two hours up to six hours and after 24 hours growth. Bacterial numbers (CFU/mL) were made at these time points by serial dilution, plating, and direct colony counts. For MS analysis, 2 mL of each sample were harvested and spun at 500 × *g* for one minute to pellet blood cells. The supernatant was transferred to a clean tube and spun at 4000 × *g* to generate a bacterial pellet. Extraction of pelleted cells and MS analysis were carried out by our standardized protocol described below.

### Analysis of mixed samples

Mixture samples were acquired from pure cultures that were mixed or from bacteria co-cultured together. Additionally, bacterial cultures were grown and extracted separately, and pure extracts mixed together to better control for the amount of lipids in these samples. Bacterial enumeration for all samples and MS analysis were performed. Cells were harvested to generate a pellet and extracted by the standardized protocol described below.

### Small-scale ammonium isobutyrate lipid extraction

To obtain samples of the highest purity that generated high-quality mass spectra for inclusion in and validation of the library, strains were streaked onto lysogeny broth agar plates to obtain pure colonies. Overnight liquid cultures (1 single colony in 1–5 mL lysogeny broth at 37 °C) were harvested, processed and converted to lipid A/LTA by an optimized hot ammonium isobutyrate-based protocol originally described by El Hamidi *et al*.^23^. Briefly, bacterial pellets were treated with a 5:3 mixture of 70% (v/v) isobutyric acid/1 M ammonium hydroxide (250 µL/150 µL) (Sigma-Aldrich, St. Louis MO) and incubated at 100 °C for 30 to 45 minutes. Reactions were spun down at 2000 × *g* for 15 minutes to remove cell debris, and supernatants were transferred to clean tubes, combined in a 1:1 ratio of distilled water, and lyophilized overnight. The resulting dry pellets contain whole cell extracts of membrane lipids.

### MALDI-TOF

Extracts were washed twice with methanol and then resuspended in 50–200 µL of a 2:1:0.25 chloroform/methanol/water (Fisher Scientific, Waltham MA; Quality Biological, Gaithersburg MD) solvent mixture. Aliquots of 1 µL each were spotted directly onto stainless steel target plates. Mass spectra were recorded in negative ion mode by direct analysis of extracts within the lipid matrix norharmane (10 mg/mL in 2:1 v/v chloroform/methanol) (Sigma-Aldrich, St. Louis MO) using a Bruker Microflex LRF MALDI-TOF MS (Bruker Daltonics Inc., Billerica MA) operated in reflectron mode. The instrument is equipped with a 337 nm nitrogen laser, and analyses were performed at 39.5% global intensity. Typically, 900–1000 laser shots were summed to acquire each mass spectrum.

### Statistical analyses

Mass spectra were imported and analyzed using R (v3.3.1) with the MALDIquant (v1.16.2) and MALDIquantForeign (v0.10) R packages^36^. Intensities of each mass spectrum were square root transformed and smoothed with a 21 point Savitzky-Golay filter^37^. Mass spectra were base line corrected using the SNIP method over 60 iterations, then aligned^38^. *Bona fide* glycolipid ions were detected by selecting ions with an 8.0 signal-to-noise ratio, with noise calculated by 21 point median absolute deviation and binning matching mass peaks between spectra using a 0.5 *m/z* tolerance. Hierarchical clustering was performed using the pvclust (v2.0–0) R package^39^. A pairwise dot product was calculated from unit vectors created from a mass list of ions from each mass spectrum, containing the normalized ion intensity of each ion.

For generation of consensus spectra for discrimination between colistin-susceptible and resistant *A*. *baumannii* and *K*. *pneumoniae*, 50% of replicate mass spectra were chosen at random to build a consensus spectra and the remaining 50% were held out as a test set. Detected ions for the chosen spectra were normalized relative to the base peak and summed together to yield a consensus spectrum. Dot products between each of the test set mass spectra and the consensus spectra for specific species were calculated as described above.

### Software analyses

The MALDI Biotyper (Bruker Daltonics Inc., Billerica MA) is a data acquisition and analysis software workflow, which incorporates Bruker FLEX series mass spectrometers including the Bruker Microflex LRF. The Biotyper was trained to recognize glycolipid mass spectra by adjusting parameters within the framework of the software to ID bacteria as follows: 1) Adjusting mass range to 1000 through 2400 *m/z* and 2) Altering peak picking thresholds to at least 10 ions to account for simpler spectra and adjusting signal-to-noise ratio to 8. Mass spectra were filtered prior to analysis in Biotyper using the filterSpectra.R script, which is available in the GitHub repository (provided below in Code availability). Parameters were optimized to reject the maximum amount of low-quality spectra: passing mass spectra were required to contain 10 ions with signal-to-noise ratios greater than or equal to 8.0. Mass spectra were loaded into the Biotyper OC software. A Main SPectra (MSP) was created for each species or phenotype (susceptible *v*. resistant, growth temperature), which is a representation of the mass spectra within each group. The number of mass spectral replicates per MSP can be found in Supplementary Tables S1, S2, and S4. For identification of *A*. *baumannii* and *K*. *pneumoniae*, 70% of randomly selected mass spectra from a single organism were used to create an MSP and the remaining 30% were tested against the generated library. The MALDI Biotyper picks peaks by Spectra Differentiation filter and generates mass lists for a test sample that are then compared by pattern matching algorithm to mass lists of all MSPs in the reference database. The software then outputs a list of the top ten identifications based on the highest log scores calculated from these comparisons. We opted to use the preprogrammed confidence log score value criteria of the software (2.0 to 3.0 = positive identification; 1.7 to 1.999 = probable identification; 0 to 1.699 = unreliable identification).

### Data availability

All data generated or analyzed during this study are included in this published article (and its Supplementary Information files).

### Code availability

All scripts used for this analysis are freely available and can be found in the following GitHub repository: https://github.com/wfondrie/ESKAPE_Glycolipid_MS.

## Electronic supplementary material

Supplemental Material
