# Supplementary material for: Identification of the ESKAPE pathogens by mass spectrometric analysis of microbial membrane glycolipids
Source: Sci Rep. 2017 Jul 25;7:6403. doi: 10.1038/s41598-017-04793-4 (PMC5526941; doi:10.1038/s41598-017-04793-4)
Supplement: Supplementary file 1 — Supplemental Material [file 41598_2017_4793_MOESM1_ESM.pdf]

Identification of the ESKAPE pathogens by mass spectrometric analysis of microbial membrane glycolipids - Supplementary Materials

Lisa M. Leung[a], William E. Fondrie[b], Yohei Doi[c], J. Kristie Johnson[d], Dudley K. Strickland[b], Robert K. Ernst[a]*, and David R. Goodlett[e]*

[a]*Department of Microbial Pathogenesis, University of Maryland, Baltimore MD 21201*;

[b]*Center for Vascular and Inflammatory Diseases, University of Maryland, Baltimore MD 21201*;

[c]*Division of Infectious Diseases, University of Pittsburgh, Pittsburgh PA 15261*;

[d]*Department of Pathology, University of Maryland, Baltimore MD 21201;*

[e]*Department of Pharmaceutical Sciences, University of Maryland, Baltimore MD 21201*;

* **Correspondence:** Robert K. Ernst, PhD, Email: rkernst@umaryland.edu.

David R. Goodlett, PhD, Email: dgoodlett@rx.umaryland.edu

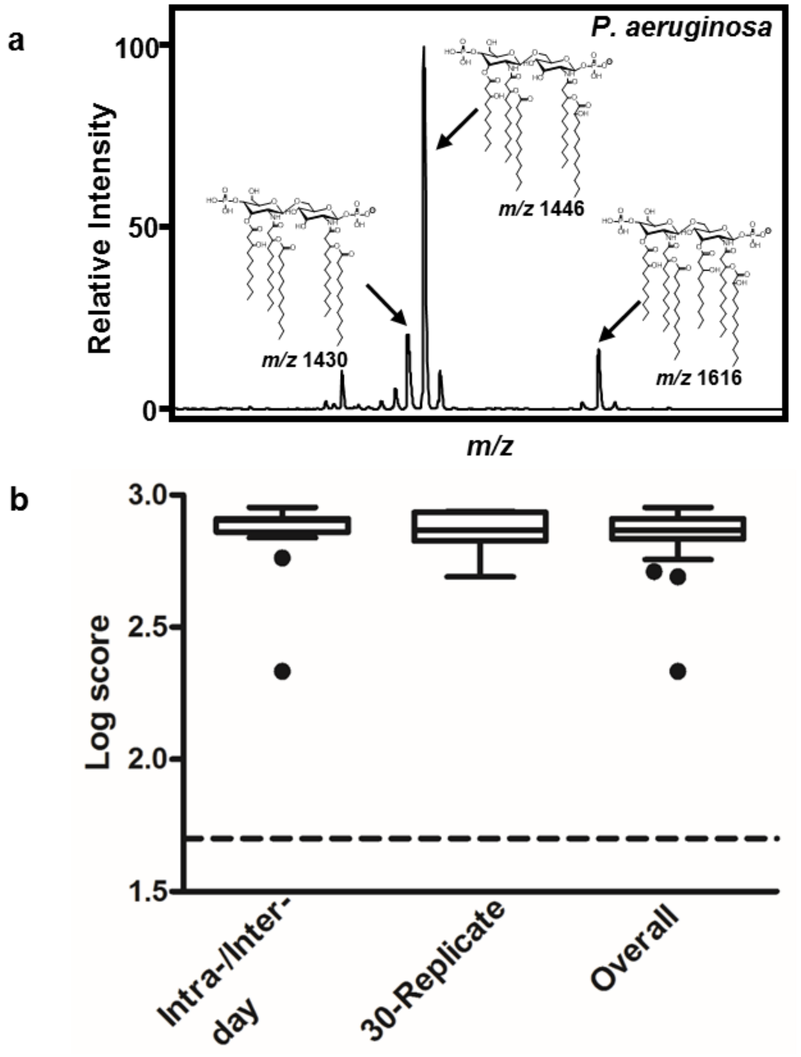

Supplementary Fig. S1. Variability determination for *P. aeruginosa*. Bacterial cultures of *P. aeruginosa* PAK laboratory strain were grown, lipids were extracted, and extracts were analyzed by MALDI-TOF to ensure reproducibility of biological and technical replicates during mass spectral library generation. (**a**) One mass spectrum is shown here with chemical structures of lipid A molecules for the annotated ions listed and (**b**) Median log confidence scores of the MALDI Biotyper when *P. aeruginosa* mass spectral replicates were compared against the glycolipid library for the Intra-/Inter-day and 30-replicate

variability assays. Dashed line indicates the threshold cutoff of a probable positive identification (=1.7).

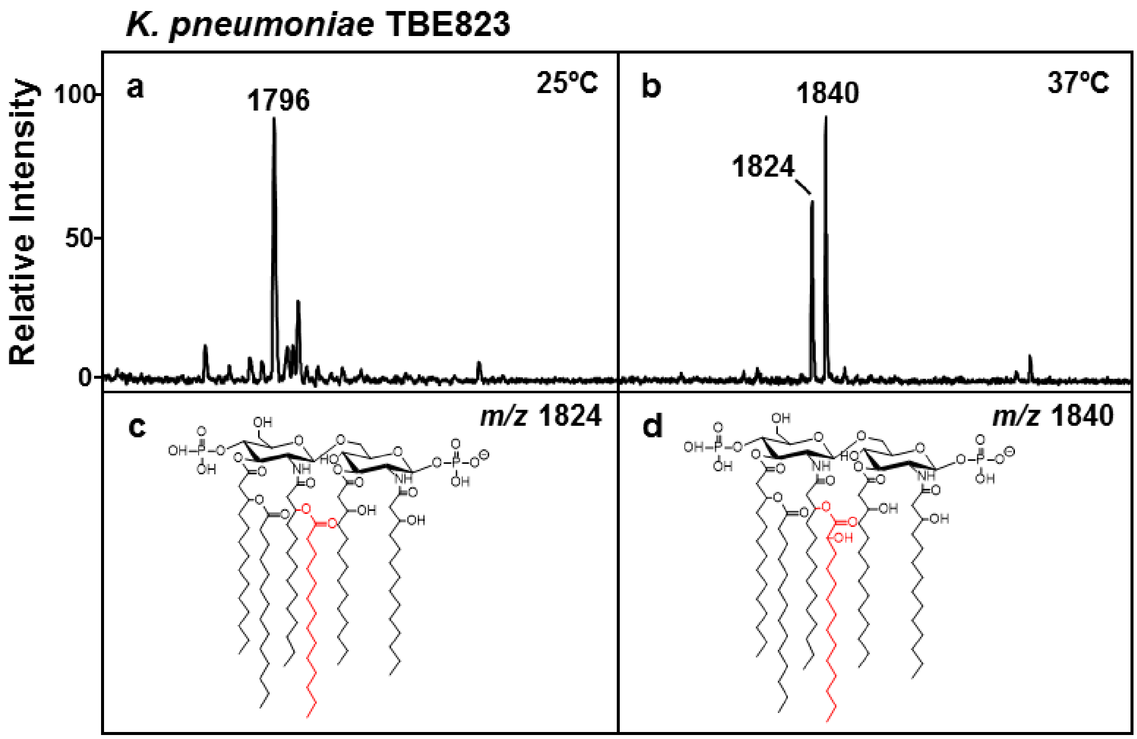

Supplementary Fig. S2. MALDI-TOF MS of *K. pneumoniae* grown at different temperatures. *K. pneumoniae* TBE823 strain grown overnight in liquid culture at 25°C (**a**) and 37°C (**b**) shows a base peak at *m/z* 1796 at 25°C that shifts to *m/z* 1824 (**c**) and 1840 (**d**) as the predominate ions at 37°C corresponding to transacylation and hydroxylation events, respectively, of the original hexa-acylated lipid A structure at *m/z* 1796.

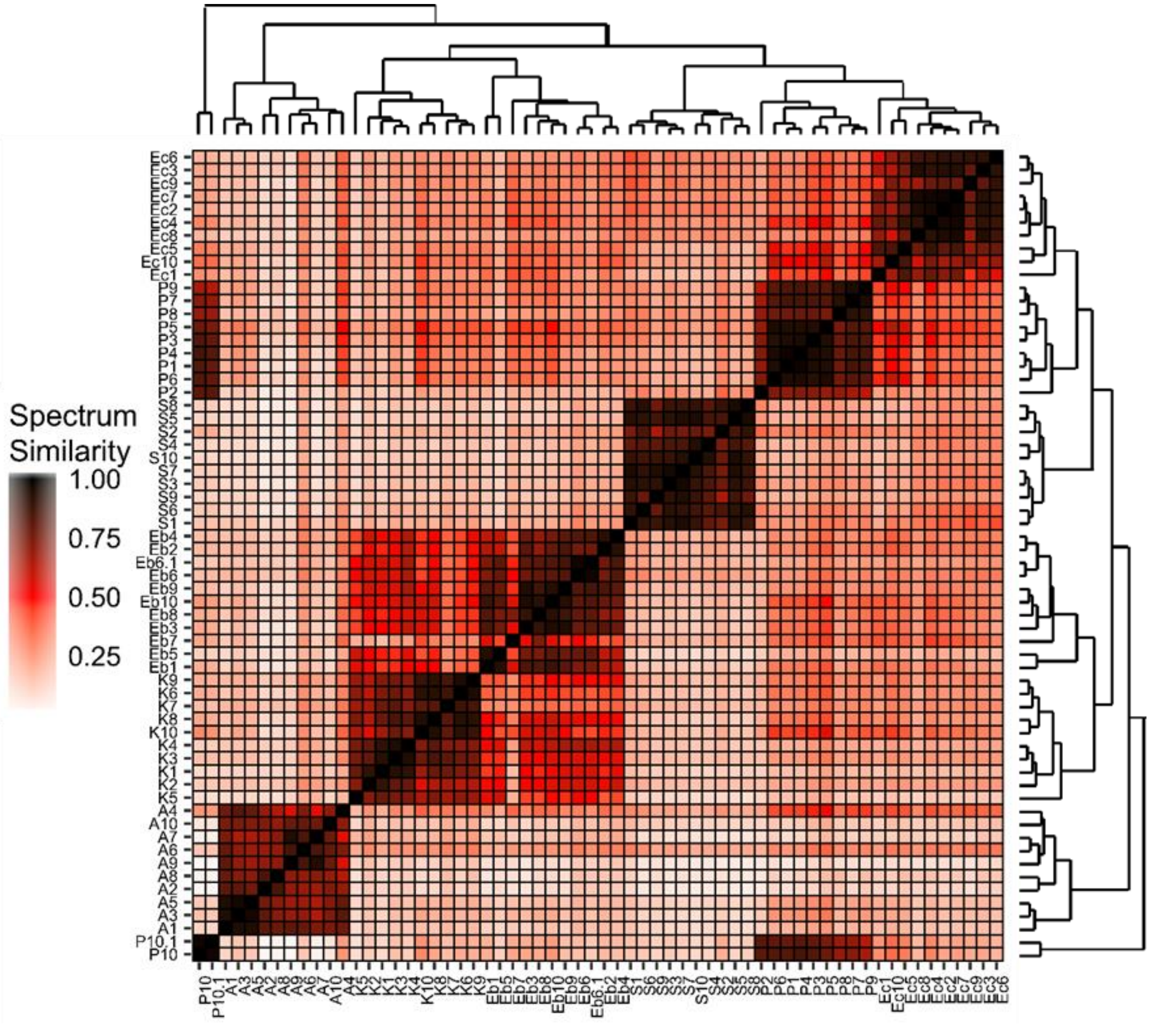

Supplementary Fig. S3. Dot product analysis with hierarchical clustering for multiple replicates per ESKAPE pathogen. Mass spectra from ten strains per ESKAPE species were compared by dot product and hierarchical clustering (Ec = *Enterococcus faecium*, S = *Staphylococcus aureus*, K = *Klebsiella pneumoniae*, A = *Acinetobacter baumannii*, P = *Pseudomonas aeruginosa*, Eb = *Enterobacter cloacae*). For *Acinetobacter* and *Klebsiella*, strains 1-5 are colistin-susceptible and 6-10 are colistin-resistant. Only *m/z* values for ions above an 8.0 signal-to-noise ratio were selected for comparison. The spectrum similarity score is the calculated dot product between mass lists from two mass spectra. A similarity score of 1.0 is an identical match (black squares). White squares represent a score of 0.0

where there is no match. Arrangement of strains in heat map reflect clustering shown in dendrogram (above).

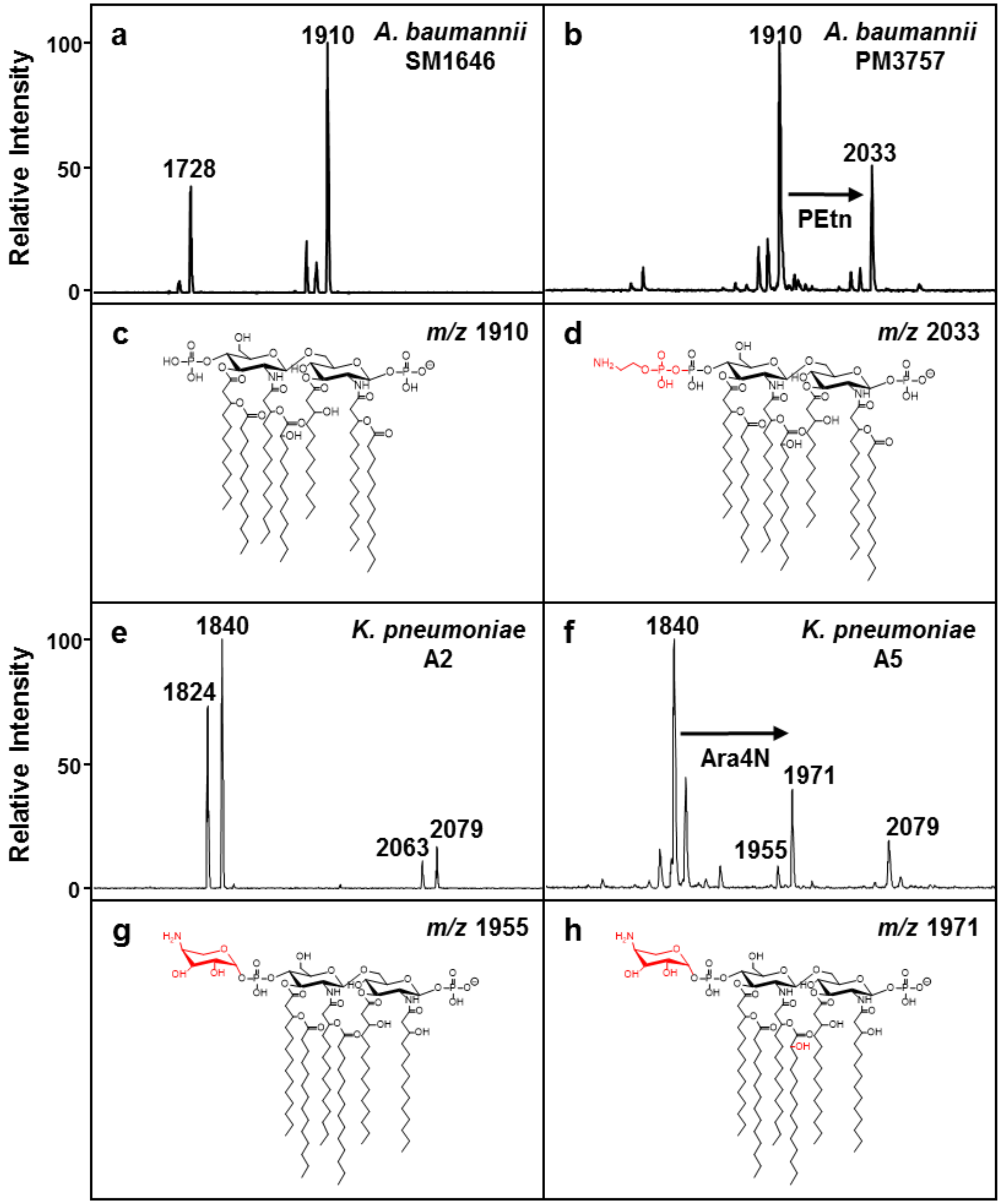

Supplementary Fig. S4. MALDI-TOF-MS of *A. baumannii* and *K. pneumoniae* with differential colistin resistance. Mass spectrum from *A. baumannii* colistin-susceptible strain SM1646 (**a**) and colistin-resistant strain PM3757 (**b**) with signature ions of hepta-acylated LA structure at *m/z* 1910 (**c**) and *m/z* 2033 (**d**) corresponding to a

phosphoethanolamine (PEtn) addition to *m/z* 1910. Mass spectrum from *K. pneumoniae* colistin-susceptible strain A2 (**e**) and colistin-resistant strain A5 (**f**) showing additional ions at *m/z* 1955 (**g**) and 1971 (**h**) corresponding to an aminoarabinose (Ara4N) addition to the base structures at *m/z* 1824 and 1840.

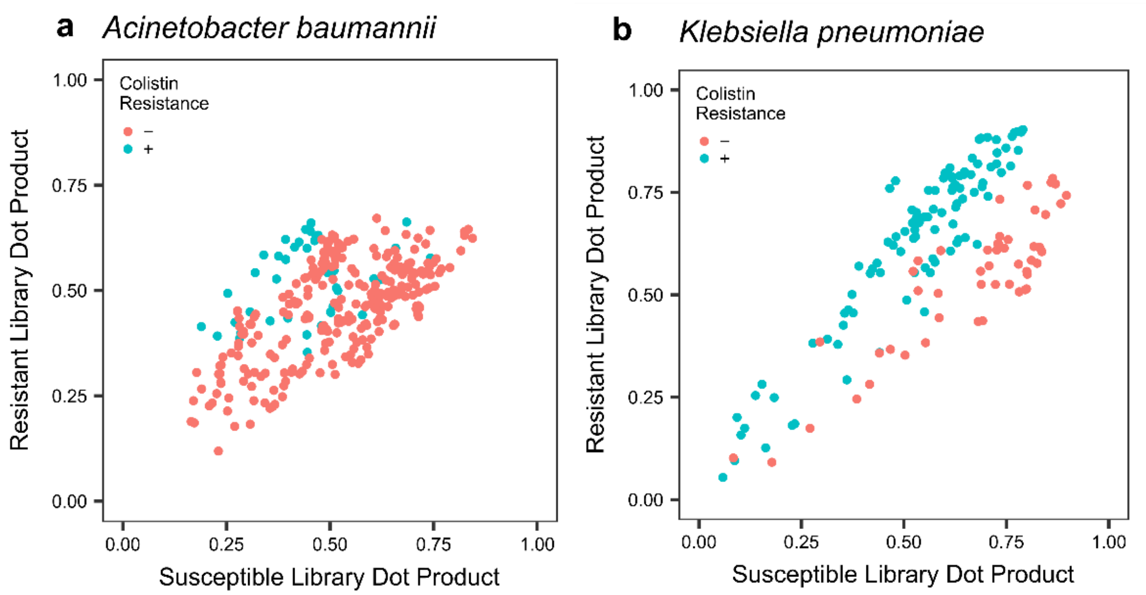

Supplementary Fig. S5. Dot product scores between library consensus spectra and test spectra partitioned by colistin resistance. Library consensus mass spectra were created from the summation of detected ions in 50% of colistin-resistant and susceptible *A. baumannii* (**a**) and *K. pneumoniae* (**b**) replicates. Dot products were calculated between each of the remaining replicates and the colistin-resistant and susceptible consensus mass spectra for each species.

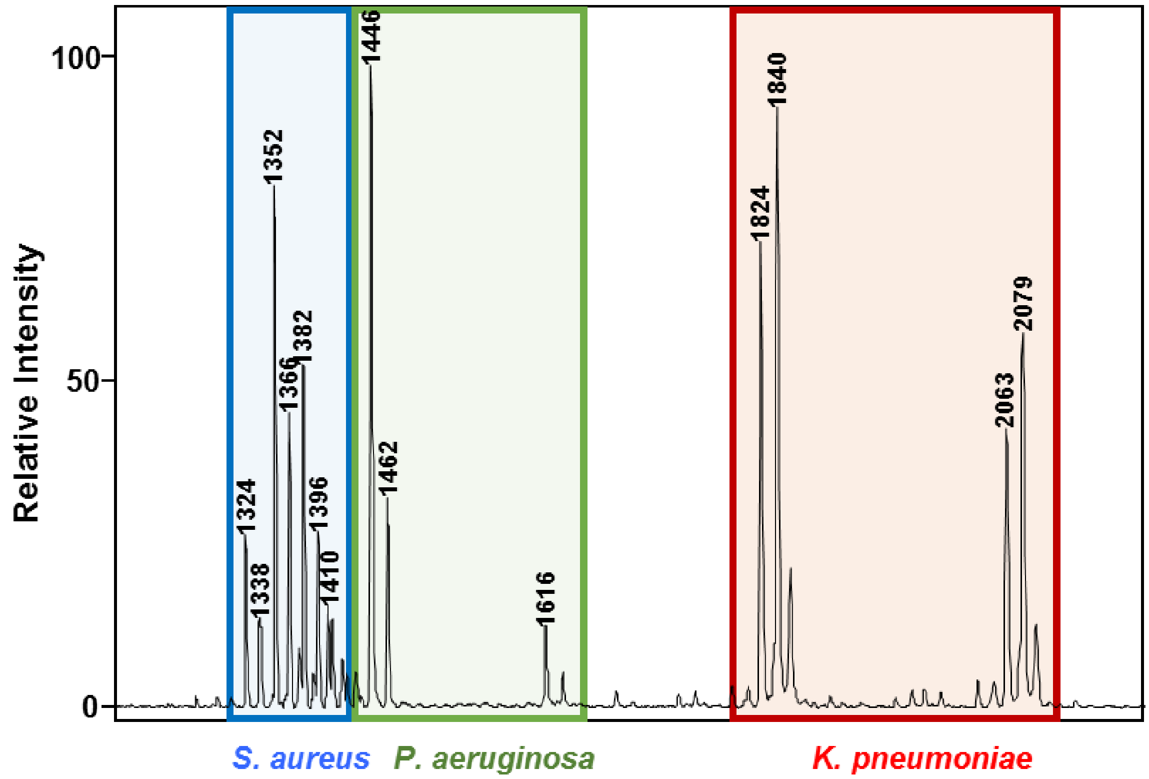

Supplementary Fig. S6. Mass spectrum from a mixed sample. *S. aureus* NRS384, *K. pneumoniae* TBE818, and *P. aeruginosa* BE399 were co-cultured, cultured separately and co-extracted, or extracted separately and mixed (shown above). Mass spectral profiles were determined. *m/z* values of ions are given and assigned to their respective organisms.

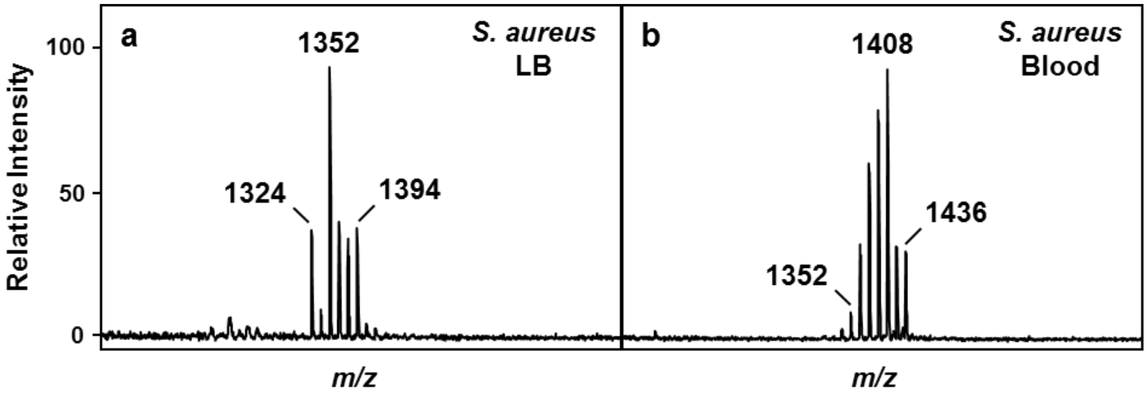

Supplementary Fig. S7. Mass spectra from *S. aureus* confirming mass shift in blood culture. *S. aureus* strain M2 grown in laboratory medium (**a**) and overnight blood culture. (**b**) A mass shift was observed from *m/z* 1352 to *m/z* 1408 as the base peak when bacteria are cultured in blood bottles. Changes in signal intensities were present at t_0 indicating immediate modifications which suggests an altered growth condition. Neighboring *m/z* values are separated from one another by 14 mass units which suggest differences of a single methylene group ($–CH_2$) and likely results from variations in fatty acid chain lengths of the DAG unit.

Supplementary Table S1. List of all ESKAPE pathogen strains used in this study.

Organism	Strain name	Map assignment	MIC	Source/resistance	Origin
Enterococcus faecium	YD1	Ec1		ICU patient	Y. Doi
	YD2	Ec2		ICU patient	Y. Doi
	YD3	Ec3		ICU patient	Y. Doi
	YD4	Ec4		ICU patient	Y. Doi
	YD5	Ec5		ICU patient	Y. Doi
	YD6	Ec6		ICU patient	Y. Doi
	YD7	Ec7		ICU patient	Y. Doi
	YD8	Ec8		ICU patient	Y. Doi
	YD9	Ec9		ICU patient	Y. Doi
	YD10	Ec10		ICU patient	Y. Doi
	YD11			VRE	Y. Doi
	YD12			VRE	Y. Doi
Staphylococcus aureus	8325-4	S1		Methicillin susceptible	M. Shirtliff
	WT	S2		Methicillin susceptible	M. Shirtliff
	RN4220	S3		Methicillin susceptible	M. Shirtliff
	RN6390	S4		Methicillin susceptible	M. Shirtliff
	Seattle 1945	S5		Methicillin susceptible	M. Shirtliff
	M2	S6		Methicillin resistant	M. Shirtliff
	NRS123	S7		Methicillin resistant	M. Shirtliff
	NRS384	S8		Methicillin resistant	M. Shirtliff
	NRS385	S9		Methicillin resistant	M. Shirtliff
	NRS484	S10		Methicillin resistant	M. Shirtliff
	NRS72			Methicillin susceptible	M. Shirtliff
	M1			Methicillin susceptible	M. Shirtliff
	NRS22			Methicillin resistant	M. Shirtliff
	NRS382			Methicillin resistant	M. Shirtliff
	NRS1			Methicillin resistant	M. Shirtliff
	NRS100			Methicillin resistant	M. Shirtliff
	NRS387			Methicillin resistant	M. Shirtliff
	DOH040			Methicillin resistant	Y. Doi
	DOH075			Methicillin resistant	Y. Doi
Klebsiella pneumoniae	A2 Obscure	K1	0.38	Abdominal fluid	Y. Doi
	B3 Bright	K2	0.25	Foley drainage	Y. Doi
	C4	K3	0.38	JP drainage	Y. Doi
	D4	K4	0.38	Blood	Y. Doi
	TBE818	K5	0.5		Y. Doi
	A5	K6	>256	BAL	Y. Doi
	B6	K7	>256	Urine	Y. Doi
	C6	K8	16	Urine	Y. Doi
	F9	K9	8		Y. Doi
	I4	K10	16	Urine	Y. Doi
	TBE812		0.25		R. Ernst
	TBE815		0.25		R. Ernst
	TBE817		0.25		R. Ernst
	TBE821		0.13		R. Ernst
	TBE823		1		R. Ernst
	TBE847		1		R. Ernst
	B3 Obscure		0.25		Y. Doi
	B5		0.25		Y. Doi
	B8		0.25		Y. Doi
	E5		0.25		Y. Doi
	F2		0.25		Y. Doi
	F8		0.38		Y. Doi
	G7		0.38		Y. Doi
	H4 Bright		0.25		Y. Doi
	I1		0.25		Y. Doi
	A8-BL13802		S		M. Hughes

Supplementary Table S1 Continued

Organism	Strain name	Map assignment	MIC	Source/resistance	Origin
Klebsiella pneumoniae	A9-BL12125		S		M. Hughes
	A11-BL12456		S		M. Hughes
	C8-KKBO-1		S		M. Hughes
	C9-KPB-1		S		M. Hughes
	TBE805		>64		R. Ernst
	TBE806		>128		R. Ernst
	TBE810		>256		R. Ernst
	TBE811		>256		R. Ernst
	TBE813		>64		R. Ernst
	TBE814		>256		R. Ernst
	TBE820		>64		R. Ernst
	TBE824		>256		R. Ernst
	TBE827		>256		R. Ernst
	B9		>256		Y. Doi
	C3		128		Y. Doi
	C5		128		Y. Doi
	C8		64		Y. Doi
	D1		16		Y. Doi
	D7		256	BAL	Y. Doi
	E6		8		Y. Doi
	F3		128		Y. Doi
	H5		64		Y. Doi
	I2		>256		Y. Doi
	A1-BL849		R		M. Hughes
	A2-BA3783		R		M. Hughes
	A3-BU19801		R		M. Hughes
	A4-BA2664		R		M. Hughes
	A5-BL8800		R		M. Hughes
	A6-BA2880		R		M. Hughes
	A7-MS84		R		M. Hughes
	C14-KKBO-1 mut_1		R		M. Hughes
	C5-KKBO-4		R		M. Hughes
	C6-KPB-2		R		M. Hughes
Acinetobacter baumannii	EAS011	A1	1	BAL	Y. Doi
	MWH019	A2	1	BAL	Y. Doi
	PM3757	A3	0.5	Urine	Y. Doi
	TBE0122	A4	2	BAL	R. Ernst
	YD14	A5	1	Sputum	Y. Doi
	EAS004	A6	>128	Sputum	Y. Doi
	SM1590	A7	4	Tracheal aspirate	Y. Doi
	PM3714	A8	16	Sputum	Y. Doi
	PM3839	A9	32	Sputum	Y. Doi
	PM3850	A10	64	Abscess	Y. Doi
	EAS001		0.5		Y. Doi
	EAS002		0.5		Y. Doi
	EAS005		0.5		Y. Doi
	EAS006		0.5		Y. Doi
	EAS008		0.5		Y. Doi
	EAS009		0.5		Y. Doi
	EAS010		1		Y. Doi
	EAS019		0.5		Y. Doi
	EAS025		0.5		Y. Doi
	EAS028		0.25		Y. Doi
	MWH001		0.5		Y. Doi
	MWH007		0.5		Y. Doi

Supplementary Table S1 Continued

Organism	Strain name	Map assignment	MIC	Source/resistance	Origin
Acinetobacter baumannii	MWH008		0.5		Y. Doi
	MWH009		0.5		Y. Doi
	MWH011		0.5		Y. Doi
	MWH012		1		Y. Doi
	MWH013		0.5		Y. Doi
	MWH014		0.5		Y. Doi
	MWH021		1		Y. Doi
	MWH023		1		Y. Doi
	MWH031		0.5		Y. Doi
	SM1533		0.5		Y. Doi
	SM1534		0.5		Y. Doi
	SM1536		0.5		Y. Doi
	SM1537		0.5		Y. Doi
	SM1538		1		Y. Doi
	SM1539		0.5		Y. Doi
	SM1540		1		Y. Doi
	SM1544 # 1		1		Y. Doi
	SM1544 # 2		0.5		Y. Doi
	SM1545		0.5		Y. Doi
	SM1549		0.5		Y. Doi
	SM1551		0.5		Y. Doi
	SM1560		0.5		Y. Doi
	SM1561		1		Y. Doi
	SM1562		1		Y. Doi
	SM1565		0.5		Y. Doi
	SM1566		0.5		Y. Doi
	SM1567		0.5		Y. Doi
	SM1568		0.5		Y. Doi
	SM1569		0.5		Y. Doi
	SM1571		0.5		Y. Doi
	SM1572		0.5		Y. Doi
	SM1574		1		Y. Doi
	SM1577		0.5		Y. Doi
	SM1578		0.5		Y. Doi
	SM1579		1		Y. Doi
	SM1580		1		Y. Doi
	SM1584		1		Y. Doi
	SM1587		1		Y. Doi
	SM1594		0.5		Y. Doi
	SM1596		0.5		Y. Doi
	SM1599		0.5		Y. Doi
	SM1600		0.5		Y. Doi
	SM1601		0.5		Y. Doi
	SM1602		0.5		Y. Doi
	SM1603		0.5		Y. Doi
	SM1604		0.5		Y. Doi
	SM1607		0.5		Y. Doi
	SM1608		0.5		Y. Doi
	SM1610		0.5		Y. Doi
	SM1627		0.5		Y. Doi
	SM1629		0.5		Y. Doi
	SM1632		1		Y. Doi
	SM1636		0.5		Y. Doi
	SM1639		1		Y. Doi
	SM1645		0.5		Y. Doi
	SM1658		0.5		Y. Doi

Supplementary Table S1 Continued

Organism	Strain name	Map assignment	MIC	Source/resistance	Origin
Acinetobacter baumannii	SM1660		1		Y. Doi
	SM1662		1		Y. Doi
	SM1665		0.5		Y. Doi
	SM1670		0.5		Y. Doi
	SM1672		0.5		Y. Doi
	SM1673		0.5		Y. Doi
	SM1675		0.5		Y. Doi
	SM1679		0.5		Y. Doi
	SM1680		0.5		Y. Doi
	SM1684		0.5		Y. Doi
	SM1685		0.5		Y. Doi
	SM1686		0.5		Y. Doi
	SM1687		0.5		Y. Doi
	SM1688		1		Y. Doi
	SM1689		1		Y. Doi
	PM3632		1		Y. Doi
	PM3636		2		Y. Doi
	PM3638		1		Y. Doi
	PM3640		1		Y. Doi
	PM3641		1		Y. Doi
	PM3642		0.5		Y. Doi
	PM3643		1		Y. Doi
	PM3647		1		Y. Doi
	PM3648		0.5		Y. Doi
	PM3651		1		Y. Doi
	PM3652		1		Y. Doi
	PM3654		0.5		Y. Doi
	PM3654a		0.5		Y. Doi
	PM3658		0.5		Y. Doi
	PM3661		1		Y. Doi
	PM3663		1		Y. Doi
	PM3664		1		Y. Doi
	PM3665		0.5		Y. Doi
	PM3667		1		Y. Doi
	PM3669		1		Y. Doi
	PM3672		1		Y. Doi
	PM3680		1		Y. Doi
	PM3682		1		Y. Doi
	PM3685		1		Y. Doi
	PM3693		1		Y. Doi
	PM3694		1		Y. Doi
	PM3696		1		Y. Doi
	PM3698		0.5		Y. Doi
	PM3700		1		Y. Doi
	PM3701		0.5		Y. Doi
	PM3716		1		Y. Doi
	PM3718		0.5		Y. Doi
	PM3719		1		Y. Doi
	PM3720		0.5		Y. Doi
	PM3721		1		Y. Doi
	PM3722		1		Y. Doi
	PM3723		0.5		Y. Doi
	PM3724		0.5		Y. Doi
	PM3725		2		Y. Doi
	PM3726		0.5		Y. Doi
	PM3728		0.5		Y. Doi

Supplementary Table S1 Continued

Organism	Strain name	Map assignment	MIC	Source/resistance	Origin
Acinetobacter baumannii	PM3731		0.5		Y. Doi
	PM3735		0.5		Y. Doi
	PM3740		0.5		Y. Doi
	PM3741		0.5		Y. Doi
	PM3742		0.5		Y. Doi
	PM3743		0.5		Y. Doi
	PM3748		0.5		Y. Doi
	PM3749		0.5		Y. Doi
	PM3752		0.25		Y. Doi
	PM3753		0.25		Y. Doi
	PM3754		0.25		Y. Doi
	PM3762		0.5		Y. Doi
	PM3769		0.5		Y. Doi
	PM3776		0.5		Y. Doi
	PM3778		0.5		Y. Doi
	PM3786		0.5		Y. Doi
	PM3790		0.5		Y. Doi
	PM3793		0.5		Y. Doi
	PM3795		1		Y. Doi
	PM3796		1		Y. Doi
	PM3797		1		Y. Doi
	PM3798		0.5		Y. Doi
	PM3801		1		Y. Doi
	PM3802		1		Y. Doi
	PM3809		1		Y. Doi
	PM3811		1		Y. Doi
	PM3813		1		Y. Doi
	PM3817		0.5		Y. Doi
	PM3820		1		Y. Doi
	PM3821		1		Y. Doi
	PM3823		0.5		Y. Doi
	PM3824		0.5		Y. Doi
	PM3827		0.5		Y. Doi
	PM3828		0.5		Y. Doi
	PM3829		1		Y. Doi
	PM3831		0.5		Y. Doi
	PM3833		0.5		Y. Doi
	PM3834		0.5		Y. Doi
	PM3836		1		Y. Doi
	PM3844		1		Y. Doi
	PM3845		1		Y. Doi
	PM3848		1		Y. Doi
	PM3865		0.5		Y. Doi
	PM3892		0.5		Y. Doi
	PM3897		0.5		Y. Doi
	PM3898		1		Y. Doi
	PM3905		1		Y. Doi
	PM3907		0.5		Y. Doi
	PM3908		1		Y. Doi
	PM3918		0.5		Y. Doi
	PM3929		0.5		Y. Doi
	PM3930		0.5		Y. Doi
	ATCC17978		1		Y. Doi
	TBE779		1		R. Ernst
	TBE1020		1		R. Ernst
	TBE1023		2		R. Ernst

Supplementary Table S1 Continued

Organism	Strain name	Map assignment	MIC	Source/resistance	Origin
Acinetobacter baumannii	TBE1025		2		R. Ernst
	TBE1029		2		R. Ernst
	TBE1015		>128	Urine	R. Ernst
	TBE1021		>256		R. Ernst
	TBE1027		>256		R. Ernst
	TBE1031		>256		R. Ernst
	EAS003		>128		Y. Doi
	SM1589		4		Y. Doi
	SM1591		4		Y. Doi
	SM1592		4		Y. Doi
	SM1622		4		Y. Doi
	SM1624		4		Y. Doi
	SM1637		64		Y. Doi
	SM1682		32		Y. Doi
	SM1683		32		Y. Doi
	PM3633		>128		Y. Doi
	PM3849		8		Y. Doi
	PM3855		>128		Y. Doi
	PM3859		32		Y. Doi
	PM3860		>128		Y. Doi
	PM3911		8		Y. Doi
	PM3914		16		Y. Doi
Pseudomonas aeruginosa	ATCC31482	P1			ATCC
	ATCC55734	P2			ATCC
	ATCC700888	P3			ATCC
	ATCC700829	P4			ATCC
	V022	P5			R. Ernst
	BE402	P6			R. Ernst
	YD15	P7			Y. Doi
	TRPA179	P8			Y. Doi
	ATCC47085	P9			ATCC
	PAO1	P10			R. Ernst
	ATCC43495				ATCC
	V015				R. Ernst
	V055				R. Ernst
	V093				R. Ernst
	TRPA087				Y. Doi
Enterobacter cloacae	FN2541	Eb1		ICU patient	J.K. Johnson
	FN2542	Eb2		ICU patient	J.K. Johnson
	FN2543	Eb3		ICU patient	J.K. Johnson
	YDC590	Eb4		Bronchial wash	Y. Doi
	YDC673	Eb5		Perigastric fluid	Y. Doi
	YDC665	Eb6		BAL	Y. Doi
	YDC470	Eb7			Y. Doi
	YDC482	Eb8			Y. Doi
	YDC541	Eb9			Y. Doi
	YDC612	Eb10		BAL	Y. Doi
	FN2532			ICU patient	J.K. Johnson
	YDC560-1				Y. Doi
	YDC611				Y. Doi

Supplementary Table S2. List of species, strains and mass spectra in Biotyper glycolipid library.

Class	Organism	Strain	# replicates	Origin
Gram-negative bacteria	*Bordetella pertussis*	Wild-type	4	R. Ernst
	Brucella abortus	2308	2	R. Ernst
	Brucella melitensis	16M	1	R. Ernst
	Brucella suis	1330	1	R. Ernst
	Burkholderia cenocepacia	CEP0790	7	R. Ernst
		N3	2	R. Ernst
		ATCC 17759	6	ATCC
	Burkholderia multivorans	ATCC 17616	9	ATCC
	Citrobacter spp.	M12	1	Y. Doi
		M17	1	Y. Doi
		BL17316	8	M. Hughes
	Enterobacter aerogenes	ENF 10856	6	BD Biosciences
		ENF 11218	7	BD Biosciences
		ENF 11237	10	BD Biosciences
		YDC497	6	Y. Doi
	Enterobacter sakazakii	YD255	3	Y. Doi
		YD256	2	Y. Doi
	Escherichia coli	K12	46	R. Ernst
		ENF 18187	8	BD Biosciences
		ATCC 25922	2	ATCC
		YDC107	3	Y. Doi
		YD626	2	Y. Doi
	Franciscella novicida	U112	39	R. Ernst
	Francisella tularensis holarctica	LVS	2	R. Ernst
	Klebsiella oxytoca	ENF 3950	9	BD Biosciences
		ENF 4321	9	BD Biosciences
		ENF 11686	9	BD Biosciences
	Legionella bozemanii	4648	4	T. McNealy
	Legionella pneumophila	ATCC LA-1	3	ATCC
		ATCC Concord 3	3	ATCC
		ATCC Bloom 2	4	ATCC
		ATCC 33152	4	ATCC
		5099	3	T. McNealy
		4632	3	T. McNealy
		1782	3	T. McNealy
	Legionella wadsworthii	5706	3	T. McNealy
	Morganella morgannii	YDC562	6	Y. Doi
		YDC700	6	Y. Doi
		YDC721	6	Y. Doi
		YDC723	5	Y. Doi
	Porphyromonas gingivalis	W50	13	R. Ernst
	Proteus mirabilis		8	R. Ernst
		YDC672-1	6	Y. Doi
		YDC714	6	Y. Doi
		NO-051/03	6	M. Hughes
	Providencia rettgeri		9	Y. Doi
	Providencia stuartii	YDC737	8	Y. Doi
		YDC672-2	6	Y. Doi
		YD257	6	Y. Doi
	Pseudomonas fluorescens	ATCC BAA-477	6	R. Ernst
	Pseudomonas fluorescens	BE561	1	R. Ernst
	Pseudomonas putida	ATCC 700007	2	R. Ernst
		6732-1	3	R. Ernst
		ATCC 49128	1	R. Ernst
		BE560	1	R. Ernst
	Pseudomonas stutzeri	TBE589	1	R. Ernst

Supplementary Table S2 Continued

Class	Organism	Strain	# replicates	Origin
Gram-negative bacteria	*Salmonella minnesota*	R595	10	R. Ernst
	Salmonella typhimurium	CS339	9	R. Ernst
	Serratia marcescens	SM3	13	Y. Doi
		SM4	10	Y. Doi
		SM5	10	Y. Doi
		SM8	1	Y. Doi
		SM11	10	Y. Doi
		SM12	10	Y. Doi
		SM13	7	Y. Doi
		YDC507	3	Y. Doi
		YDC563	4	Y. Doi
		YDC583	3	Y. Doi
		YDC591	3	Y. Doi
		YDC609	4	Y. Doi
		YDC629	3	Y. Doi
		YDC639	3	Y. Doi
		YDC647	3	Y. Doi
		YDC719	3	Y. Doi
		5	7	M. Hughes
	Stenotrophomonas maltophilia	CF2	5	R. Ernst
	Yersinia enterolitica	CS080	10	R. Ernst
	Yersinia pestis	KIM6-pCDI-pgm	10	R. Ernst
		KIM6+ (Bliska)	2	R. Ernst
	Yersinia pseudotuberculosis	01:b	6	R. Ernst
Gram-positive bacteria	*Clostridium difficile*	630	1	R. Ernst
		uk1	1	R. Ernst
	Enterococcus faecalis	FN1	3	J.K. Johnson
		FN2	7	J.K. Johnson
		FN14	5	J.K. Johnson
		FN39	8	J.K. Johnson
		FN45	5	J.K. Johnson
		FN46	4	J.K. Johnson
		FN59	8	J.K. Johnson
		FN69	6	J.K. Johnson
		FN71	6	J.K. Johnson
		FN77	7	J.K. Johnson
		YD258	3	Y. Doi
		YD259	3	Y. Doi
		YD260	3	Y. Doi
		YD261	2	Y. Doi
	Staphylococcus epidermidis	POS 10235	6	BD Biosciences
	Staphylococcus haemolyticus	POS 10866	6	BD Biosciences
		POS 8764	3	BD Biosciences
	Staphylococcus lugdunensis	POS 8659	3	BD Biosciences
		POS 10768	1	BD Biosciences
	Streptococcus mitis	POS 4489	6	BD Biosciences
	Streptococcus mitis	POS 5586	6	BD Biosciences
	Streptococcus mutans	POS 1260	6	BD Biosciences
		POS 5593	5	BD Biosciences
	Streptococcus pneumoniae	POS 6289	4	BD Biosciences
		POS 6892	3	BD Biosciences
		POS 10164	2	BD Biosciences
	Streptococcus sanguinis	POS 4696	6	BD Biosciences
		POS 5589	6	BD Biosciences
Fungi	*Candida albicans*	YST 1032	3	BD Biosciences
		YST 1369	4	BD Biosciences

Supplementary Table S2 Continued

Class	Organism	Strain	# replicates	Origin
Fungi	*Candida albicans*	YST 1862	4	BD Biosciences
		YD262	3	Y. Doi
		YD263	3	Y. Doi
	Candida glabrata	YD264	4	Y. Doi
		YD265	3	Y. Doi
	Candida krusei	YD266	3	Y. Doi
	Candida parapsilosis	YD267	3	Y. Doi
**TOTAL**	**44**	**117**	**655**	

Supplementary Table S3. Similarity scores for small heat map dot product comparison.

	Enterococcus faecium	*Staphylococcus aureus* (MRSA)	*Staphylococcus aureus*	*Klebsiella pneumoniae* 37°C	*Klebsiella pneumoniae* *	*Klebsiella pneumoniae* 25°C	*Acinetobacter baumannii*	*Acinetobacter baumannii* *	*Pseudomonas aeruginosa* 25°C	*Pseudomonas aeruginosa* 37°C	*Enterobacter cloacae* 25°C	*Enterobacter cloacae* 37°C
Enterobacter cloacae 37°C	0.31	0.19	0.18	0.16	0.30	0.77	0.23	0.17	0.31	0.21	0.54	1.00
Enterobacter cloacae 25°C	0.16	0.10	0.09	0.43	0.27	0.82	0.10	0.11	0.29	0.15	1.00	0.54
Pseudomonas aeruginosa 37°C	0.38	0.25	0.23	0.13	0.23	0.21	0.15	0.14	0.55	1.00	0.15	0.21
Pseudomonas aeruginosa 25°C	0.43	0.25	0.23	0.25	0.38	0.33	0.24	0.21	1.00	0.55	0.29	0.31
Acinetobacter baumannii *	0.11	0.11	0.09	0.12	0.21	0.18	0.75	1.00	0.21	0.14	0.11	0.17
Acinetobacter baumannii	0.18	0.09	0.09	0.11	0.21	0.16	1.00	0.75	0.24	0.15	0.10	0.23
Klebsiella pneumoniae 25°C	0.25	0.13	0.11	0.35	0.30	1.00	0.16	0.18	0.33	0.21	0.82	0.77
Klebsiella pneumoniae *	0.27	0.19	0.18	0.79	1.00	0.30	0.21	0.21	0.38	0.23	0.27	0.30
Klebsiella pneumoniae 37°C	0.14	0.11	0.10	1.00	0.79	0.35	0.11	0.12	0.25	0.13	0.43	0.16
Staphylococcus aureus	0.15	0.95	1.00	0.10	0.18	0.11	0.09	0.09	0.23	0.23	0.09	0.18
Staphylococcus aureus (MRSA)	0.17	1.00	0.95	0.11	0.19	0.13	0.09	0.11	0.25	0.25	0.10	0.19
Enterococcus faecium	1.00	0.17	0.15	0.14	0.27	0.25	0.18	0.11	0.43	0.38	0.16	0.31

*** (*) indicates colistin resistance**

Supplementary Table S4. MALDI Biotyper results for identifying *K. pneumoniae* and *A. baumannii*.

Organism		# strains	# replicates	Mean confidence log score	% positive sub-species identification	% positive species identification
Klebsiella pneumoniae	Colistin-susceptible	26	96	2.528	**100.0**	**100.0**
	Colistin-resistant	34	220	2.536	**69.2**	**87.7**
Acinetobacter baumannii	Colistin-susceptible	188	555	2.270	**55.9**	**77.2**
	Colistin-resistant	25	93	2.615	**92.3**	**92.3**

*** 30% of strains were used as testing sets to determine ID rates of accuracy**
† Positive identifications were determined to be the top-scoring organism and a log score > 1.7
